# Extent and predictors of guideline-directed medical therapy optimization during cardiac rehabilitation in patients with heart failure

**DOI:** 10.1016/j.ijcrp.2026.200579

**Published:** 2026-01-09

**Authors:** Corentin Nicolas, Nicolas Girerd, Kevin Duarte, Olivier Huttin, Karim Djaballah, Jerome Felloni, Guillaume Baudry, Luca Monzo

**Affiliations:** a CHRU de Nancy, Centre d'Investigation Clinique Plurithématique 1433, INSERM, Université de Lorraine, INSERM U1116 - DCAC, and F-CRIN INI-CRCT (Cardiovascular and Renal Clinical Trialists), Nancy, France; b CHRU de Nancy, Department of Cardiology, Université de Lorraine, Nancy, France

**Keywords:** Heart failure, Cardiac rehabilitation, Guideline-directed medical therapy, Optimization

## Abstract

**Background:**

Cardiac rehabilitation (CR) may offer a structured framework for guideline-directed medical therapy (GDMT) optimization, but its real-world impact is uncertain. We aimed to quantify GDMT optimization and identify its clinical predictors during CR.

**Methods:**

This retrospective single-centre study included patients hospitalized for acute HF with reduced (HFrEF) or mildly reduced (HFmrEF) ejection fraction who subsequently underwent first inpatient or ambulatory CR at Nancy University Hospital (2021–2024). Changes in GDMT optimization were evaluated using the HF prescription and the KCMO scores and expressed as adjusted standardized differences (ASD). Multivariable linear regression identified independent predictors of optimization.

**Results:**

Among the 106 patients included (84 % HFrEF, mean age 59 years; 75 % male), baseline GDMT use was high, but doses were suboptimal. During CR, significant uptitration occurred across all major drug classes, including angiotensin receptor–neprilysin inhibitors (ASD: +22 %), beta-blockers (ASD: +21 %), mineralocorticoid receptor antagonists (ASD: +43 %), and sodium–glucose cotransporter 2 inhibitors (ASD: +45 %) (all p < 0.001). Overall GDMT optimization improved significantly, as evidenced by increases in both the KCMO (ASD: +39.9) and HF prescription (ASD: +1.27) scores (both p < 0.001), with consistent effects across inpatient and ambulatory settings. In multivariable analysis, higher loop-diuretic dose and prior treatment by HF specialists were associated with less optimization, whereas hypertension predicted greater intensification.

**Conclusions:**

Cardiac rehabilitation after HF hospitalization promoted substantial GDMT optimization, especially in hypertensive patients. Higher loop-diuretic dose at admission predicted less optimization, suggesting that minimizing diuretic doses may ease GDMT titration.

## Highlights

• Cardiac rehabilitation enables structured GDMT optimization after acute HF.
• Significant uptitration observed across all major HF drug classes.
• Hypertensive patients achieved the greatest GDMT optimization.
• Higher diuretic dose and prior specialist care associated with less optimization opportunity.

## 1 Introduction

Early initiation and up-titration of guideline-directed medical therapy (GDMT), including renin–angiotensin system (RAS) inhibitors (angiotensin converting enzyme inhibitors [ACEi], angiotensin receptor blockers [ARB], and angiotensin-receptor neprilysin inhibitor [ARNI]), beta-blockers, mineralocorticoid receptor antagonists (MRAs), and sodium-glucose co-transporter 2 (SGLT2) inhibitors, provides substantial prognostic benefit in patients with heart failure (HF) [1], with greater dose intensity linked to lower mortality and hospitalization [2]. Yet, registry data show that only a minority of patients achieve target doses in routine care, as many remain undertreated due to comorbidities, side-effect concerns, or insufficient monitoring [3,4].

Cardiac rehabilitation (CR) is a multidisciplinary intervention that combines supervised exercise training, therapeutic education, psychosocial support, and medical optimization [5]. Beyond its established role in improving quality of life and functional status, CR may also contribute to reducing all-cause and HF-related hospitalizations [6]. The structured follow-up and close monitoring during CR provide an ideal context for the safe titration and optimization of GDMT, addressing issues of tolerance and adherence that often hinder treatment intensification in outpatient care.

Despite these theoretical advantages, the extent to which GDMT optimization is achieved during CR, and the factors predicting successful titration, have been insufficiently investigated. We therefore aim to assess changes in GDMT and identify clinical predictors of treatment intensification in patients with HF with reduced (HFrEF) and mildly reduced (HFmrEF) ejection fraction involved in a CR program.

## 2 Methods

This retrospective study included patients hospitalized for acute HF (AHF) with left ventricular ejection fraction <50 % who were included for the first time in a CR program between 2021 and 2024 at Nancy University Hospital. Rehabilitation was performed as conventional inpatient or in the ambulatory setting. The primary objective was to assess changes in HF therapy during the rehabilitation program and identify patient characteristics associated with treatment intensification.

Demographic data, clinical history, biological parameters, and echocardiographic measurements, were collected at admission in the CR program. Individuals with missing treatment data at admission or discharge from CR program were excluded. Exercise training during the CR program consisted of supervised aerobic and resistance sessions, delivered in accordance with French national recommendations [7]. Exercise intensity and modalities were individualized at the discretion of the treating rehabilitation physician, based on baseline exercise capacity, clinical status, haemodynamic parameters, and patient tolerance, and were progressively adapted throughout the program. Heart rate and blood pressure were routinely monitored during sessions, with additional ECG monitoring when clinically indicated. GDMT optimization during the CR program was performed by the multidisciplinary rehabilitation team following a pragmatic, guideline-informed approach based on clinical evaluation, without a predefined titration algorithm. Heart failure treatment was assessed using two composite measures: the Kansas City Medication Optimization (KCMO) score and the HF prescription score.

### 2.1 KCMO score

The KCMO score quantifies both use and intensity of GDMT [8]. For each eligible drug class (RAS inhibitors, beta-blockers, MRAs, SGLT2 inhibitors), the total daily dose (prescribed dose × frequency) is divided by the guideline target dose, as indicated in the latest HF guidelines [9]. For SGLT2 inhibitors, since only one dose is approved [10], any prescription was considered equivalent to the target dose (i.e., ratio = 1.0). Resulting ratios are capped at 1.0 (100 %) and averaged across all eligible classes (classes with documented contraindication or intolerance are excluded from the denominator). The average is then multiplied by 100, yielding a 0–100 scale where 100 indicates target dosing in all eligible classes.

### 2.2 HF prescription score

The HF prescription score was calculated at entry and discharge from CR using five therapeutic classes: RAS inhibitors, ARNI, beta-blocker, MRA, and SGLT2 inhibitor. For each class, the prescribed daily dose was expressed as a fraction of the guideline-recommended target dose (0–1, capped at 1 if higher than target) [9]. The score was then obtained by summing these fractions, yielding a range from 0 (no therapy) to 5 (all classes at target dose). Accordingly, a one-point increase in the HF prescription score corresponds to one GDMT drug class reaching its target dose. For ARNI, any prescribed dose was counted both as a RAS inhibitor and ARNI, thus contributing twice to the total score.

### 2.3 Statistical analysis

Continuous variables were summarized as mean ± standard deviation (SD) and median (interquartile range, IQR), and categorical variables as counts and percentages. Baseline characteristics were compared between inpatient and outpatient CR using the Kruskal–Wallis test for continuous and the chi-square or Fisher's exact test for categorical variables.

Changes in HF therapies (drug doses and composite scores) between admission and discharge were evaluated in the overall cohort and by CR type using linear regression adjusted for baseline values. Results are reported as admission and discharge means, adjusted mean differences (AMD), and 95 % confidence intervals (CIs). Associations between patient characteristics and change in HF prescription score (delta) were explored by multivariable linear regression adjusted for baseline score, age, and sex. Variables with p < 0.20 in adjusted analyses were candidates for the final model, which was built using stepwise forward selection (p < 0.05 for entry), forcing age, sex, and baseline score. This approach was used to limit model complexity relative to the modest sample size and to identify the most relevant predictors in an exploratory framework, while retaining key clinical covariates. Pairwise interactions between selected predictors were tested and retained if significant.

Multicollinearity was evaluated using variance inflation factors (VIFs), with all values < 2, indicating no relevant collinearity. Linearity of continuous predictors was assessed using restricted cubic splines (3–5 knots at conventional quantiles) in models adjusted for age, sex, and baseline HF prescription score; when evidence of non-linearity was observed, variables were categorized using clinically meaningful thresholds. Residual diagnostics (residuals vs. fitted values, normal Q–Q plots, scale–location plots, and residuals vs. leverage plots) did not reveal major violations of linearity, normality, or homoscedasticity. Model goodness-of-fit was assessed using the coefficient of determination (R^2^) and adjusted R^2^. No imputation was deemed necessary given the low proportion of missing data and the absence of missingness for HF treatment variables at admission and discharge. Beta coefficients are presented with 95 % CIs. Analyses were performed with R (version 4.1.2), and two-sided p < 0.05 was considered statistically significant.

## 3 Results

### 3.1 Study population

A total of 106 HF patients (16 % HFmrEF, 84 % HFrEF) entering for the first time a CR program was included in the primary analysis (81 in inpatient and 25 in ambulatory CR). Baseline characteristics of the overall study population are summarized in Table 1.

**Table 1:** Baseline characteristics of the study population.

	Missing	Overall (n = 106)
Demographic and clinical		
Age, years	0	58.8 ± 12.0
Sex, n. (%)		
Male	0	79 (74.5)
Female		27 (25.5)
BMI, kg/m^2^	0	26.9 ± 6.6
HR, bpm	0	74.7 ± 13.5
Systolic BP, mmHg	2	110.0 ± 20.8
Diastolic BP, mmHg	2	67.3 ± 12.8
NYHA class, n. (%)		
I	0	60 (56.6)
II		21 (19.8)
III		20 (18.9)
IV		5 (4.7)
Ischaemic HF aetiology, n. (%)	0	60 (56.6)
Admission source, n. (%)		
Cardiac ICU	0	17 (16.0)
Ward		58 (54.7)
Outpatient clinic		31 (29.2)
**Comorbidities**		
COPD, n. (%)	1	8 (7.5)
Hypertension, n. (%)	0	53 (50.0)
Diabetes, n. (%)	0	27 (25.5)
Smoking habit, n. (%)		
Previous smokers	2	30 (28.8)
Current smokers		25 (24.0)
Non-smokers		49 (47.1)
**Echocardiography**		
LVEF, %	0	32.1 ± 8.7
LVEF ≤40 %, n. (%)	0	89 (84.0)
PAPs, mmHg	20	31.6 ± 20.4
TAPSE, mm	10	17.0 ± 4.9
**Blood**		
Creatinine, μmol/L	14	101.9 ± 40.8
eGFR, mL/min/1.73m^2^	14	76.0 ± 25.3
Haemoglobin, g/dL	12	13.3 ± 2.4
Sodium, mmol/L	15	140.9 ± 2.7
Potassium, mmol/L	15	4.2 ± 0.5
NT pro-BNP, pg/mL	13	3158 (1252; 5988)
**Treatment**		
ACEi/ARB, n. (%)	0	28 (26.4)
ARNI, n. (%)	0	71 (67.0)
ACEi/ARB/ARNI, n. (%)	0	99 (93.4)
Beta-blockers, n. (%)	0	96 (90.6)
MRA, n. (%)	0	86 (81.1)
SGLT2 inhibitors, n. (%)	0	86 (81.1)
Devices, n. (%)		
CRT	0	11 (10.4)
ICD		41 (38.7)
PMK		12 (11.3)

Abbreviations: ACEi, angiotensin-converting enzyme inhibitor; ARB, angiotensin receptor blocker; ARNI, angiotensin receptor–neprilysin inhibitor; BMI, body mass index; BP, blood pressure; COPD, chronic obstructive pulmonary disease; CRT, cardiac resynchronization therapy; CR, cardiac rehabilitation; eGFR, estimated glomerular filtration rate; HF, heart failure; HR, heart rate; ICD, implantable cardioverter defibrillator; ICU, intensive care unit; LVEF, left ventricular ejection fraction; MRA, mineralocorticoid receptor antagonist; NT-proBNP, N-terminal pro–B-type natriuretic peptide; NYHA, New York Heart Association; PAPs, pulmonary artery systolic pressure; PMK, pacemaker; SGLT2, sodium–glucose co-transporter 2; TAPSE, tricuspid annular plane systolic excursion.

The mean age was 59 ± 12 years, most patients were male (75 %), and the mean body mass index (BMI) was 27 kg/m^2^. The predominant HF aetiology was ischemic (57 %). Median N-terminal pro–B-type natriuretic peptide (NT-proBNP) was 3158 (IQR 1252; 5988) pg/mL, while the mean left ventricular ejection fraction (LVEF) was 32 %. Comorbidities were frequent in this cohort, with nearly half of the patients having hypertension (49 %), around one-quarter diabetes (26 %), and over half history of smoking habit (53 %).

The mean duration of inpatient CR program was 18.2 ± 7.3 days, whereas ambulatory CR consisted of 19.3 ± 4.6 one-day sessions. No patient missed a session due to safety concerns. Baseline characteristics were largely comparable between patients enrolled in inpatient and ambulatory CR. Nevertheless, patients referred to inpatient CR displayed features suggestive of greater clinical vulnerability, including a higher resting heart rate (77 vs. 70 bpm) and median NT-proBNP levels (3435 vs. 720 pg/mL), together with lower diastolic blood pressure (66 vs. 72 mmHg), TAPSE (17 vs. 19), and haemoglobin levels (13 vs. 15 g/dL). No significant differences were observed regarding NYHA class, LVEF, systolic blood pressure, admission source, baseline comorbidities, or use of GDMT (Supplemental Table 1).

### 3.2 Baseline treatment and evolution

**Fig. 1:**
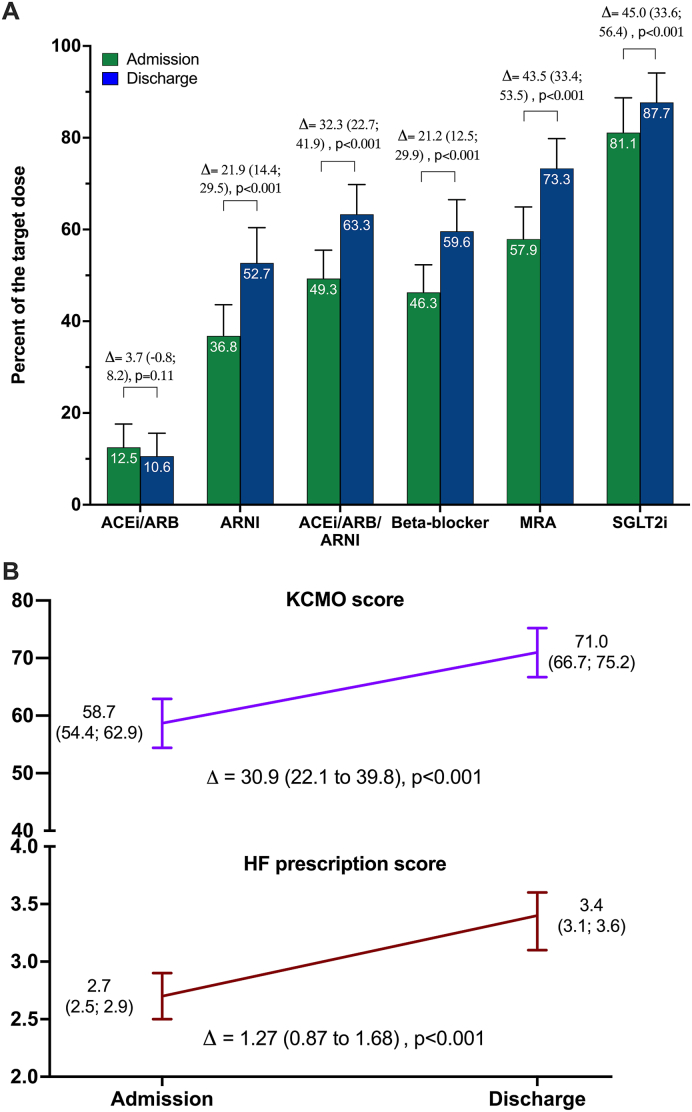
**Optimization of guideline-directed medical therapy during cardiac rehabilitatio**n. A) Mean proportion of the target dose achieved for each drug class at admission and discharge, with 95 % confidence intervals. **B)** Changes in the KCMO score and the HF prescription score between admission and discharge. Values are shown as adjusted mean differences (Δ) with 95 % confidence intervals and p-value between admission and discharge. Estimates were derived from linear mixed-effects regression models with a random patient effect and adjustment for baseline values. Abbreviations: ACEi, angiotensin-converting enzyme inhibitor; ARB, angiotensin receptor blocker; ARNI, angiotensin receptor–neprilysin inhibitor; HF, heart failure; KCMO, Kansas City Medical Optimization score; MRA, mineralocorticoid receptor antagonist; SGLT2i, sodium–glucose co-transporter 2 inhibitor.

Overall, at the initiation of CR program, the study population was treated with a high uptake of GDMT. A RAS inhibitor was prescribed in 93 % of patients (26 % ACEi/ARB and 67 % ARNI), while beta-blockers were used in 91 % of patients. MRAs and SGLT2 inhibitors were each prescribed in 81 % of patients (Table 1). Despite the broad use of GDMT, treatment intensity was suboptimal, with average doses at admission corresponding to only 13 % of the target ACE inhibitors or ARB, 37 % for ARNI, 46 % for beta-blockers, and 58 % for MRAs (Fig. 1A). The baseline KCMO score was 58.7 and the HF prescription score was 2.7 (Fig. 1B).

During CR program, significant uptitration was observed across all major drug classes. The AMD increases in the proportion of target dose achieved was 21.9 % for ARNI, 21.2 % for beta-blockers, 43.5 % for MRA, and 45.0 % for SGLT2 inhibitors (all p < 0.001). In contrast, ACEi/ARBs showed a non-significant increase (p = 0.11), likely reflecting treatment optimization achieved through switches to ARNI rather than further dose escalation. However, when considered as a composite category, RAS inhibitors (ACEi/ARB/ARNI) increased by 32.3 % (p < 0.001) (Fig. 1A). These changes translated into significant improvements in both the KCMO (ASD: 30.9 [95 % CI, 22.1; 39.8], p < 0.001) and HF prescription score (ASD: 1.3 [95 % CI, 0.9; 1.7], p < 0.001) (Fig. 1B). Subgroup analyses revealed consistent results between inpatient and ambulatory programs (Supplemental Table 2).

### 3.3 Determinants of therapeutic optimization

In univariable analyses, a higher loop diuretic dose at admission (>80 mg/day), impaired renal function, higher pulmonary systolic pressure, and prior management by a HF specialist were associated with lower likelihood of GDMT up-titration, whereas the presence of hypertension and higher systolic blood pressure were associated with more favourable treatment intensification. No significant differences were observed according to the type of CR program (inpatient vs. ambulatory) (Supplemental Table 3).

In multivariable analyses, higher loop diuretic dose at discharge remained independently associated with smaller improvement in the HF prescription score, with a graded effect observed for doses of 1–80 mg/day (β −0.41, [95 % CI −0.70 to −0.13]) and >80 mg/day (β −1.04, [95 % CI −1.39 to −0.70]) compared with no loop diuretics. Prior in-hospital management by a HF specialist was also associated with lower score improvement (β −0.51, [95 % CI −0.77 to −0.24]), whereas hypertension was positively associated with greater treatment optimization (β 0.33, [95 % CI 0.06 to 0.59]) (Table 2).

**Table 2:** Multivariable model for the change in heart failure prescription score between entry and discharge from cardiac rehabilitation program.

	Beta (95 % confidence interval)∗	p-value
HF prescription score at admission	−0.25 (−0.37 to −0.13)	<0.0001
Age, for 10 years increase	−0.06 (−0.17 to 0.06)	0.35
Sex, female	−0.19 (−0.49 to 0.12)	0.23
LD dose at discharge		<0.0001
None	Ref.	–
1–80 mg	−0.41 (−0.70 to −0.13)	0.005
>80 mg	−1.04 (−1.39 to −0.70)	<0.0001
Treated by HF specialists	−0.51 (−0.77 to −0.24)	0.0003
Hypertension	0.33 (0.06–0.59)	0.015
	Multiple R^2^: 0.463; Adjusted R^2^: 0.424	

Abbreviations: HF, heart failure; LD, loop diuretics; LVEF, left ventricular ejection fraction.

## 4 Discussion

In this real-world cohort of patients with HFrEF and HFmrEF, participation in a structured CR program was associated with clinically meaningful implementation of evidence-based HF treatment, regardless of inpatient or ambulatory setting. From a practical perspective, high-dose loop diuretics and prior management by a HF specialist were both associated with smaller improvements in the HF prescription score, corresponding to roughly one and half a drug class fewer reaching target dose, respectively. Conversely, hypertension was associated with a modest but clinically meaningful increase in treatment intensification, equivalent to approximately one-third of an additional drug class achieving target dose. Our findings support the evolving concept of CR as more than a purely functional or rehabilitative intervention. The multidimensional nature of CR, combining structured exercise training, close clinical monitoring, and patient education, may offer an ideal environment to overcome common barriers to GDMT intensification after acute HF, such as limited follow-up, hemodynamic concerns, and perceived frailty.

The early post-acute phase has long been recognized as a key window for GDMT initiation. Several randomized trials have demonstrated that the in-hospital introduction of GDMT is both feasible and safe in stabilized patients [[11], [12], [13]], with therapeutic benefits emerging rapidly [14,15], thereby providing early protection during the high-risk post-discharge period. The STRONG-HF (Safety, Tolerability and Efficacy of Rapid Optimization, Helped by NT-proBNP Testing, of Heart Failure Therapies) trial further reinforced this concept, showing that rapid and intensive GDMT optimization following HF hospitalization led to improved symptoms, quality of life, and reduced 180-day mortality or HF readmission compared with usual care, without major safety concerns [1]. However, it should be noted that the tight follow-up and resource-intensive framework implemented in STRONG-HF have been criticized as likely difficult to reproduce in everyday clinical practice due to organizational constraints. In this context, CR programs address implementation gaps highlighted in recent ESC guidelines [9] and may represent an attractive and pragmatic alternative for GDMT uptitration beyond hospital discharge, bridging the transition between the acute and chronic phases, often marked by therapeutic inertia, while alleviating the burden on outpatient HF clinics. A recent Cochrane meta-analysis of 60 randomized trials confirmed that participation in CR meaningfully reduces HF hospitalizations and improves quality of life across delivery models, even though no mortality reduction was observed [6]. Notably, no safety concerns associated with participation in exercise-based CR were identified, consistent with long-standing evidence supporting its favourable profile. Despite these demonstrated benefits, CR interventions remain markedly underused, with participation rates below 30 % worldwide [5].

Although baseline GDMT use was high in our cohort, dose intensity remained suboptimal, a pattern consistent with the therapeutic inertia widely documented in contemporary HF registries [4]. The magnitude of GDMT increase observed in our cohort was substantial (RAS inhibitors +32 %, beta-blockers +21 % for, MRAs +43 %, SGLT2 inhibitors +45 %) and compares favourably with contemporary trial data. In the CONNECT-HF (Care Optimization Through Patient and Hospital Engagement Clinical Trial for Heart Failure) trial, which evaluated a comprehensive hospital- and post-discharge quality-improvement intervention including clinician education, audit-and-feedback, and structured follow-up tools, pharmacological optimization over 12 months was minimal. Indeed, the proportion of patients achieving at least half of the target dose increased by only 1.5 % for beta-blockers, 0.06 % for RAS inhibitors, and 4.6 % for MRAs in the intervention arm compared with usual care [16]. This contrast underscores the persistent challenge of achieving meaningful medication optimization in routine practice, even within structured system-level programmes.

However, even within the CR setting, the degree of optimization was limited by some clinical factors. As expected, a higher loop-diuretic dose was associated with less GDMT uptitration, whereas hypertension predicted greater optimization. This likely reflects the fact that hypertensive patients generally tolerate higher target doses, whereas those requiring higher diuretic regimens often have lower blood pressure, a condition often viewed as a barrier to further optimization [17]. Yet, as underscored in a recent HFA consensus document, low blood pressure in the absence of symptoms should not deter GDMT intensification [18]. Additionally, the relationship between higher loop-diuretic requirements and limited optimization is consistent with the STRONG-HF framework, in which greater congestion was frequently associated with the need for higher diuretic doses and impaired haemodynamic stability [1], thereby limiting GDMT uptitration. Prior follow-up by HF specialists was paradoxically associated with less GDMT optimization. Indeed, this likely reflects a ceiling effect, as these patients were already optimised to maximum tolerated doses during hospitalization [19], leaving limited room for further escalation.

Alongside the key role of CR programs in GDMT implementation, other structured approaches such as nurse-led monitoring [20], early post-discharge reviews [21], and the use of telemedicine and remote physiological monitoring [22,23], have also been shown to facilitate safe and timely GDMT escalation. These strategies may help clinicians detect intolerance early, anticipate decompensation, and support patients throughout titration, potentially reducing dropouts and improving adherence, and should therefore be considered a key component of post-acute HF care programs [24].

## 5 Limitations

Our study has some limitations. Its retrospective design, single-centre setting, lack of correction for multiple testing, and relatively small sample size warrant cautious interpretation, as these factors may preclude causal inference, introduce selection bias, increase the risk of spurious associations, and limit the generalizability of the findings to other healthcare systems or CR programme structures. Adverse events were not systematically collected or adjudicated, potentially reducing the ability to comprehensively assess safety and tolerability. However, no patient discontinued or missed CR sessions because of treatment-related adverse events, suggesting that GDMT uptitration during CR was well tolerated in this setting. Unmeasured confounders, such as frailty, adherence, or socioeconomic factors, may have influenced the observed associations. Adequate control for these factors will be essential in future studies to ensure the robustness and accuracy of the findings. The use of stepwise selection in the multivariable models may increase the risk of overfitting; therefore, these results should be considered exploratory and hypothesis-generating. Finally, our study did not include an evaluation of hard clinical endpoints and therefore cannot determine whether the observed GDMT optimization translates into improved long-term outcomes.

## 6 Conclusions

Post-acute CR was associated with substantial GDMT optimization in HFrEF/HFmrEF patients, underscoring its potential as a key setting to address undertreatment in HF. Optimization was most pronounced in hypertensive patients, whereas higher loop-diuretic dose at admission and prior HF-specialist care predicted less intensification. Future prospective multicentre studies are needed to determine whether structured GDMT optimization within CR programs translates into improved long-term clinical outcomes.

## CRediT authorship contribution statement

**Corentin Nicolas:** Writing – original draft, Investigation, Data curation. **Nicolas Girerd:** Writing – review & editing, Supervision, Project administration, Methodology, Conceptualization. **Kevin Duarte:** Methodology, Formal analysis. **Olivier Huttin:** Writing – review & editing, Investigation. **Karim Djaballah:** Writing – review & editing, Investigation. **Jerome Felloni:** Writing – review & editing, Investigation. **Guillaume Baudry:** Writing – review & editing, Investigation. **Luca Monzo:** Writing – review & editing, Writing – original draft, Supervision, Conceptualization.

## Funding

None.
