# Supplementary material for: Extent and predictors of guideline-directed medical therapy optimization during cardiac rehabilitation in patients with heart failure
Source: Int J Cardiol Cardiovasc Risk Prev. 2026 Jan 9;28:200579. doi: 10.1016/j.ijcrp.2026.200579 (PMC12824905; doi:10.1016/j.ijcrp.2026.200579)
Supplement: Multimedia component 1 [file mmc1.docx]

# **Supplemental Table 1** Baseline characteristics of the study population by type of cardiac rehabilitation program

|  | **Inpatient CR (n=81)** | **Outpatient CR (n=25)** | **p-value*** |
| --- | --- | --- | --- |
| **Demographic and clinical** |  |  |  |
| Age, years | 59.3 ± 11.9 | 57.2 ± 12.4 | 0.32 |
| Sex, n. (%) |  |  | 0.60 |
| Male | 59 (72.8) | 20 (80.0) |  |
| Female | 22 (27.2) | 5 (20.0) |  |
| BMI, kg/m² | 26.6 ± 6.7 | 27.7 ± 6.1 | 0.29 |
| HR, bpm | 76.5 ± 13.1 | 68.8 ± 13.2 | 0.014 |
| Systolic BP, mmHg | 109.8 ± 20.5 | 110.7 ± 22.4 | 1.00 |
| Diastolic BP, mmHg | 65.9 ± 12.6 | 72.2 ± 12.4 | 0.026 |
| NYHA class, n. (%) |  |  | 0.57 |
| I | 46 (56.8) | 14 (56.0) |  |
| II | 14 (17.3) | 7 (28.0) |  |
| III | 17 (21.0) | 3 (12.0) |  |
| IV | 4 (4.9) | 1 (4.0) |  |
| Ischaemic HF aetiology, n. (%) | 47 (58.0) | 13 (52.0) | 0.65 |
| Admission source, n. (%) |  |  | 0.077 |
| Cardiac ICU | 14 (17.3) | 3 (12.0) |  |
| Ward | 48 (59.3) | 10 (40.0) |  |
| Outpatient clinic | 19 (23.5) | 12 (48.0) |  |
| **Comorbidities** |  |  |  |
| COPD, n. (%) | 5 (6.2) | 3 (12.0) | 0.39 |
| Hypertension, n. (%) | 44 (54.3) | 9 (36.0) | 0.17 |
| Diabetes, n. (%) | 21 (25.9) | 6 (24.0) | 1.00 |
| Smoking habit, n. (%) |  |  | 0.95 |
| Previous smokers | 23 (28.7) | 7 (29.2) |  |
| Current smokers | 20 (25.0) | 5 (20.8) |  |
| Non-smokers | 37 (46.2) | 12 (50.0) |  |
| **Echocardiography** |  |  |  |
| LVEF, % | 31.3 ± 8.5 | 34.6 ± 8.9 | 0.077 |
| LVEF ≤40%, n. (%) | 70 (86.4) | 19 (76.0) | 0.22 |
| PAPs, mmHg | 31.4 ± 20.3 | 32.5 ± 21.2 | 0.74 |
| TAPSE, mm | 16.5 ± 4.6 | 19.4 ± 5.5 | 0.021 |
| **Blood** |  |  |  |
| Creatinine, µmol/L | 101.5 ± 40.7 | 104.3 ± 42.7 | 0.72 |
| eGFR, mL/min/1.73m² | 75.7 ± 25.0 | 77.6 ± 28.3 | 0.75 |
| Haemoglobin, g/dL | 13.0 ± 2.3 | 14.6 ± 2.4 | 0.012 |
| Sodium, mmol/L | 140.9 ± 2.9 | 140.5 ± 1.0 | 0.59 |
| Potassium, mmol/L | 4.3 ± 0.5 | 3.9 ± 0.3 | 0.013 |
| NT pro-BNP, pg/mL | 3435 (1588; 6263) | 720 (318; 2900) | 0.002 |
| **Treatment** |  |  |  |
| ACEi/ARB, n. (%) | 22 (27.2) | 6 (24.0) | 1.00 |
| ARNI, n. (%) | 52 (64.2) | 19 (76.0) | 0.34 |
| ACEi/ARB/ARNI, n. (%) | 74 (91.4) | 25 (100.0) | 0.19 |
| Beta-blockers, n. (%) | 72 (88.9) | 24 (96.0) | 0.45 |
| MRA, n. (%) | 65 (80.2) | 21 (84.0) | 0.78 |
| SGLT2i, n. (%) | 66 (81.5) | 20 (80.0) | 1.00 |
| Devices, n. (%) |  |  |  |
| CRT | 11 (13.6) | 0 (0.0) | 0.063 |
| ICD | 34 (42.0) | 7 (28.0) | 0.25 |
| PMK | 9 (11.1) | 3 (12.0) | 1.00 |

*****p-value from Wilcoxon test for continuous variables or Fisher's exact for categorical variables.

Abbreviations: ACEi, angiotensin-converting enzyme inhibitor; ARB, angiotensin receptor blocker; ARNI, angiotensin receptor–neprilysin inhibitor; BMI, body mass index; BP, blood pressure; COPD, chronic obstructive pulmonary disease; CRT, cardiac resynchronization therapy; CR, cardiac rehabilitation; eGFR, estimated glomerular filtration rate; HF, heart failure; HR, heart rate; ICD, implantable cardioverter defibrillator; ICU, intensive care unit; LVEF, left ventricular ejection fraction; MRA, mineralocorticoid receptor antagonist; NT-proBNP, N-terminal pro–B-type natriuretic peptide; NYHA, New York Heart Association; PAPs, pulmonary artery systolic pressure; PMK, pacemaker; SGLT2i, sodium–glucose co-transporter 2 inhibitor; TAPSE, tricuspid annular plane systolic excursion.

# **Supplemental Table 2** Changes in heart failure treatment doses and optimization scores by cardiac rehabilitation type

|  | **Inpatient CR** **(n=81)** | **Outpatient CR** **(n=25)** | **Adjusted mean** **difference**  **(95% CI)*** | **p-value** |
| --- | --- | --- | --- | --- |
| ACEi/ARB (% of target dose) |  |  |  |  |
| Admission | 12.2 (6.3 to 18.0) | 13.5 (3.0 to 24.0) |  |  |
| Discharge | 9.3 (3.6 to 14.9) | 15.0 (4.8 to 25.2) |  |  |
| Difference | -2.93 (-8.29 to 2.42) | 1.50 (-8.14 to 11.14) | 5.02 (-4.64 to 14.68) | 0.31 |
| ARNI (% of target dose) |  |  |  |  |
| Admission | 34.6 (26.8 to 42.4) | 44.0 (30.0 to 58.0) |  |  |
| Discharge | 50.5 (41.6 to 59.3) | 60.0 (44.1 to 75.9) |  |  |
| Difference | 15.90 (9.80 to 21.99) | 16.00 (5.03 to 26.97) | 1.67 (-10.74 to 14.07) | 0.79 |
| ACEi/ARB/ARNI (% of target dose) |  |  |  |  |
| Admission | 46.8 (39.8 to 53.8) | 57.5 (44.9 to 70.1) |  |  |
| Discharge | 59.7 (52.4 to 67.0) | 75.0 (61.8 to 88.2) |  |  |
| Difference | 12.96 (6.44 to 19.48) | 17.50 (5.76 to 29.24) | 8.70 (-3.69 to 21.09) | 0.17 |
| Beta-blocker (% of target dose) |  |  |  |  |
| Admission | 44.0 (37.1 to 50.8) | 54.0 (41.7 to 66.3) |  |  |
| Discharge | 54.8 (47.1 to 62.5) | 75.0 (61.1 to 88.9) |  |  |
| Difference | 10.81 (5.25 to 16.37) | 21.00 (11.00 to 31.00) | 12.15 (0.88 to 23.42) | 0.035 |
| MRA (% of target dose) |  |  |  |  |
| Admission | 57.3 (49.2 to 65.3) | 60.0 (45.4 to 74.6) |  |  |
| Discharge | 74.4 (67.0 to 81.8) | 70.0 (56.7 to 83.3) |  |  |
| Difference | 17.13 (9.90 to 24.36) | 10.00 (-3.02 to 23.02) | -5.81 (-18.42 to 6.80) | 0.36 |
| SGLT2i (% of target dose) |  |  |  |  |
| Admission | 81.5 (72.8 to 90.2) | 80.0 (64.3 to 95.7) |  |  |
| Discharge | 86.4 (79.1 to 93.7) | 92.0 (78.9 to 105.1) |  |  |
| Difference | 4.94 (-2.04 to 11.92) | 12.00 (-0.57 to 24.57) | 6.36 (-5.32 to 18.05) | 0.28 |
| KCMO score |  |  |  |  |
| Admission | 57.4 (52.5 to 62.2) | 62.9 (54.1 to 71.6) |  |  |
| Discharge | 68.8 (64.0 to 73.6) | 78.0 (69.3 to 86.7) |  |  |
| Difference | 11.46 (7.59 to 15.33) | 15.12 (8.16 to 22.09) | 5.48 (-1.86 to 12.81) | 0.14 |
| HF prescription score |  |  |  |  |
| Admission | 2.6 (2.4 to 2.9) | 3.0 (2.5 to 3.4) |  |  |
| Discharge | 3.3 (3.0 to 3.5) | 3.7 (3.2 to 4.2) |  |  |
| Difference | 0.62 (0.43 to 0.81) | 0.76 (0.43 to 1.10) | 0.22 (-0.15 to 0.59) | 0.24 |

*Mean difference between admission and discharge, estimated using linear regression adjusted for baseline values.

Abbreviations: ACEi, angiotensin-converting enzyme inhibitor; ARB, angiotensin receptor blocker; ARNI, angiotensin receptor–neprilysin inhibitor; CI, confidence interval; HF, heart failure; KCMO, Kansas City Medical Optimization score; MRA, mineralocorticoid receptor antagonist; SGLT2i, sodium–glucose co-transporter 2 inhibitor.

# **Supplemental Table 3** Associations between baseline or discharge characteristics during cardiac rehabilitation and the change in heart failure prescription score in linear regression model adjusted for age, sex and baseline heart failure prescription

|  | **Beta (95% CI)** | **p-value** |
| --- | --- | --- |
| Age, for 10 years increase | 0.04 (-0.09 to 0.18) | 0.53 |
| Sex, female | -0.17 (-0.56 to 0.21) | 0.37 |
| BMI, kg/m² | 0.02 (-0.00 to 0.05) | 0.081 |
| Admission source |  | 0.64 |
| Cardiac ICU | Ref. |  |
| Ward | 0.20 (-0.25 to 0.66) | 0.38 |
| Outpatient clinic | 0.10 (-0.40 to 0.60) | 0.68 |
| Inpatient CR | -0.22 (-0.60 to 0.15) | 0.24 |
| Outpatient CR | 0.22 (-0.15 to 0.60) | 0.24 |
| HR, for 10 bpm increase | 0.00 (-0.12 to 0.12) | 0.96 |
| Sinus rhythm | 0.15 (-0.26 to 0.55) | 0.47 |
| Systolic BP, for 10 mmHg increase | 0.11 (0.03 to 0.19) | 0.008 |
| Diastolic BP, for 10 mmHg increase | 0.10 (-0.03 to 0.22) | 0.12 |
| NYHA class 3-4 (vs. 1-2) | -0.39 (-0.77 to -0.02) | 0.038 |
| Ischemic HF aetiology | -0.23 (-0.59 to 0.12) | 0.19 |
| **Comorbidities** |  |  |
| Hypertension | 0.35 (0.02 to 0.67) | 0.036 |
| Diabetes | 0.02 (-0.35 to 0.40) | 0.90 |
| Smoking habit |  | 0.22 |
| Previous smokers | Ref. |  |
| Current smokers | -0.38 (-0.83 to 0.07) | 0.097 |
| Non-smokers | -0.26 (-0.64 to 0.12) | 0.18 |
| COPD | -0.28 (-0.89 to 0.33) | 0.36 |
| **Devices** |  |  |
| ICD | -0.14 (-0.48 to 0.19) | 0.39 |
| PMK | -0.18 (-0.69 to 0.34) | 0.50 |
| CRT | -0.02 (-0.55 to 0.50) | 0.93 |
| LVEF ≤40% | -0.01 (-0.45 to 0.44) | 0.97 |
| TAPSE, mm | 0.01 (-0.02 to 0.05) | 0.47 |
| PAPs, for 10 mmHg increase | -0.13 (-0.22 to -0.05) | 0.003 |
| Creatinine, for 20 µmol/L increase | -0.09 (-0.17 to -0.00) | 0.049 |
| eGFR, for 10 mL/min/1.73m² increase | 0.09 (0.01 to 0.16) | 0.031 |
| Haemoglobin, g/dL | -0.01 (-0.09 to 0.07) | 0.77 |
| Loop diuretics at admission | -0.44 (-0.76 to -0.12) | 0.007 |
| Loop diuretics dose at admission |  | 0.001 |
| None | Ref. |  |
| 1-80 mg | -0.27 (-0.61 to 0.06) | 0.11 |
| >80 mg | -0.79 (-1.21 to -0.38) | 0.0003 |
| Loop diuretics at discharge | -0.71 (-1.00 to -0.41) | <0.0001 |
| Loop diuretics dose at discharge |  | <0.0001 |
| None | Ref. |  |
| 1-80 mg | -0.46 (-0.77 to -0.16) | 0.003 |
| >80 mg | -1.16 (-1.53 to -0.79) | <0.0001 |
| Treated by HF specialists | -0.59 (-0.91 to -0.28) | 0.0003 |

Abbreviations: BMI, body mass index; BP, blood pressure; COPD, chronic obstructive pulmonary disease; CRT, cardiac resynchronization therapy; CR, cardiac rehabilitation; eGFR, estimated glomerular filtration rate; HCM, hypertrophic cardiomyopathy; HF, heart failure; HR, heart rate; ICD, implantable cardioverter defibrillator; ICU, intensive care unit; LVEF, left ventricular ejection fraction; NT-proBNP, N-terminal pro–B-type natriuretic peptide; NYHA, New York Heart Association; PAPs, pulmonary artery systolic pressure; PMK, pacemaker; TAPSE, tricuspid annular plane systolic excursion.
